# Risk of intracranial hemorrhage with direct oral anticoagulants: an updated network meta-analysis of randomized controlled trials

**DOI:** 10.3389/fcvm.2026.1835091

**Published:** 2026-05-19

**Authors:** Jiana Chen, Qiaomei Chen, Chengfu Guan, Jianping Xia

**Affiliations:** Department of Pharmacy, Nanping First Hospital Affiliated to Fujian Medical University, Nanping, Fujian, China

**Keywords:** atrial fibrillation, direct oral anticoagulants, intracranial hemorrhage, network meta-analysis, venous thromboembolism

## Abstract

**Objective:**

We updated a network meta-analysis of randomized controlled trials to compare the risk of intracranial hemorrhage (ICH) between direct oral anticoagulants (DOACs) and Vitamin K Antagonists (VKAs) in detail across Venous thromboembolism and atrial fibrillation.

**Methods:**

PubMed, EMBASE, Web of Science, and the Cochrane Library databases were searched up to January 5, 2026. The incidence of ICH was investigated. Using frequentist network meta-analysis, interventions that were not compared directly could be compared indirectly by the 95% confidence interval (CI), making the search results more intuitive. Based on surface under the cumulative ranking curves (SUCRA), the relative ranking probability of each group was generated.

**Results:**

Twenty randomised controlled trials (127,267 patients) were included. Compared with apixaban, VKAs (OR: 2.40, 95% CI: 1.62–3.56) had a higher risk of bleeding, and the difference was significant. Compared with dabigatran, rivaroxaban (OR: 1.89, 95% CI: 1.18–3.04) and VKAs (OR: 2.83, 95% CI: 2.00–3.99) had a higher risk of bleeding, and the difference was significant. Compared with edoxaban, rivaroxaban (OR: 1.60, 95% CI: 1.04–2.47) and VKAs (OR: 2.39; 95% CI: 1.81–3.17) had a higher risk of bleeding, and the difference was significant. Compared with rivaroxaban, VKAs (OR: 1.50; 95% CI: 1.08–2.07) had a higher risk of bleeding, and the difference was significant. In the ranking of the cumulative probability of ICH, dabigatran (SUCRA 87.6) had the highest safety, followed by apixaban (SUCRA 68.6), edoxaban (SUCRA 67.5), rivaroxaban (SUCRA 26.1), and VKAs (SUCRA 0.2).

**Conclusions:**

All DOACs had a lower risk of ICH than VKAs. Dabigatran may be the safest choice among any anticoagulant regarding risk of ICH.

**Systematic Review Registration:**

https://www.crd.york.ac.uk/PROSPERO/view/CRD420261336668, identifier CRD420261336668.

## 1 Introduction

Direct oral anticoagulants (DOACs) have a wide therapeutic window, predictable pharmacokinetics and pharmacodynamics, no food interactions, few drug interactions and do not require routine coagulation detectio (1). The use of antithrombotic drugs is often associated with the risk of bleeding, and intracranial hemorrhage (ICH) is a possible serious complication, leading to severe disability and high mortality (2). Once it occurs, the mortality rate of patients is extremely high, and survivors often suffer from severe neurological disabilities, imposing a huge burden on individuals, families and society. Therefore, when choosing an anticoagulation strategy, weighing the antithrombotic benefits against the risk of bleeding, especially the risk of ICH, is the core of clinical decision-making. Compared with traditional vitamin K antagonists (VKAs), DOACs have been proven by a large amount of evidence to significantly reduce the risk of ICH, which is the key advantage in terms of safety and has greatly improved the prognosis and treatment confidence of patients requiring long-term anticoagulation therapy, such as those with atrial fibrillation (AF) and Venous thromboembolism (VTE) (3, 4). However, there are still conflicting results regarding the ranking of ICH risk among DOACs.

Previous meta-analyses have not provided a detailed ranking of bleeding risk for DOACs vs. VKAs stratified by different indications. Furthermore, since the publication of these prior meta-analyses, new randomized controlled trials have been published and should be incorporated. Therefore, to provide a more comprehensive and updated assessment, we used network meta-analysis (NMA) to compare the risk of ICH between different anticoagulants in patients who received anticoagulation for AF and VTE.

## 2 Methods

We performed a systematic review and meta-analysis, adhering to the Cochrane Collaboration Guidelines, and reported our findings according to the Preferred Reporting Items for Systematic Reviews and Meta-analyses (PRISMA) reporting guidelines (5). The meta-analysis was registered with the International Prospective Register of Systematic Reviews (PROSPERO identifier: CRD420261336668).

### 2.1 Data sources and searches

A systematic search was conducted in PubMed, EMBASE, Web of Science, and The Cochrane Library for relevant articles published in English prior to January 5, 2026. The search terms used were (1) Venous Thromboembolism OR Venous Thrombosis OR Pulmonary Embolism OR Vein Thromboembolism OR Vein Thrombosis OR venous thromboem* OR venous thrombos* OR deep vein thrombos* OR deep venous thrombos* OR phlebothrombos* OR pulmonary embolism OR pulmonary thromboembolism OR lung embolism OR VTE OR DVT OR PE; (2) atrial fibrillation[MeSH] OR atrial fibrillation[title/abstract] OR non-valvular atrial fibrillation[title/abstract] OR AF[title/abstract] OR NVAF[title/abstract]; (3) dabigatran OR Pradaxa OR rivaroxaban OR Xarelto OR apixaban OR Eliquis OR edoxaban OR Savaysa OR non-vitamin K antagonist oral anticoagulant* OR non-vitamin K antagonist* OR NOAC* OR direct oral anticoagulant* OR DOAC* OR novel oral anticoagulant* OR new oral anticoagulant* OR new orally active anticoagulant* OR factor Xa inhibitor* OR factor 10a inhibitor* OR factor IIa inhibitor* OR direct thrombin inhibitor*; (4) Randomized controlled trial. For the sake of comprehensiveness, we also examined the list of research references found in the database search. All literature searches and screening were independently conducted by two researchers.

### 2.2 Inclusion and exclusion criteria

The inclusion criteria were as follows: (1) comparisons between DOACs (apixaban, dabigatran, edoxaban, rivaroxaban) and the control group (VKAs); (2) patients with VTE or AF; (3) at least one ICH data was reported for both the control and experimental groups; (4) subjects were aged 18 years or older; (5) randomized controlled trials.

The exclusion criteria were as follows: (1) DOACs in combination with other antithrombotic drugs; (2) relevant ICH data not obtained.

The ICH events reported in the included RCTs were part of the definition of major bleeding or the primary safety endpoint. All these trials adopted or were compatible with the definition of the International Society on Thrombosis and Haemostasis when defining major bleeding (6). Specifically, in these trials, ICH was automatically classified as a critical-site bleeding according to the ISTH definition due to its clinical severity, thereby constituting a major bleeding event.

### 2.3 Selection of studies and data management

Two researchers independently extracted data from eligible trials, including the first author and publication year, the number of patients, age, gender ratio, the number of cases of ICH, and follow-up time. If there were any discrepancies, a third researcher would be called upon to resolve them.

### 2.4 Assessment of risk of bias of included studies and GRADE assessment

Included studies were assessed using the Risk of Bias 2 tool (RoB 2) (7) and the risk of a bias in each RCT was evaluated by two independent researchers (J.C. and Q.C.). During evaluation, disputes were resolved by the evaluation of another researcher (C.G.). The quality of evidence was assessed using the Grading of Recommendations Assessment, Development and Evaluation (GRADE) approach, which classified the quality of evidence as “high”, “moderate”, “low”, or “very low” (8).

### 2.5 Statistical analysis

In the present study, Stata (StataCorp, College Station, TX, USA) based on a frequentist framework was used for the NMA. First, the extracted data were sorted and paired, and then imported into Stata 16.0. The node size and thickness of the line in the network evidence map represent the number of patients included in the corresponding intervention and number of directly compared interventions. If the funnel chart was roughly symmetrical and the Egger test *p* > .05, there may be no publication bias in the NMA. The odds ratio (OR) and 95% confidence interval (CI) from forest plots were used to evaluate whether the difference in the incidence of bleeding was significant between the interventions. If the CI did not cross the value 1, then the result was considered significant and there was a significant difference between the two. The surface under the cumulative ranking curves (SUCRA) was used to assess the bleeding risk. The larger the area under SUCRA, the lower the risk of bleeding and the higher the safety. It is important to note that the SUCRA ranking is a relative ranking derived from the calculation of posterior probabilities based on the model. The results should be regarded as a summary description of the existing evidence rather than definitive evidence of treatment superiority. All clinical inferences must primarily be based on direct comparisons of treatment effect sizes (such as OR) and their 95% CI. The node splitting method is employed to evaluate the inconsistency between direct and indirect comparisons within each closed loop in the network. The inconsistency factor and its 95% CI will be calculated, and a statistical significance threshold of *p* < 0.10 will be used to determine if there is inconsistency. If no closed loops are formed in the network, we will state that a formal inconsistency assessment cannot be conducted and support the rationality of indirect comparisons through transitivity assessment. To examine transitivity, the population, intervention, comparison, and outcome methodology was used to assess each of the selected articles.

## 3 Result

### 3.1 Literature retrieval and screening process

**Figure 1:**
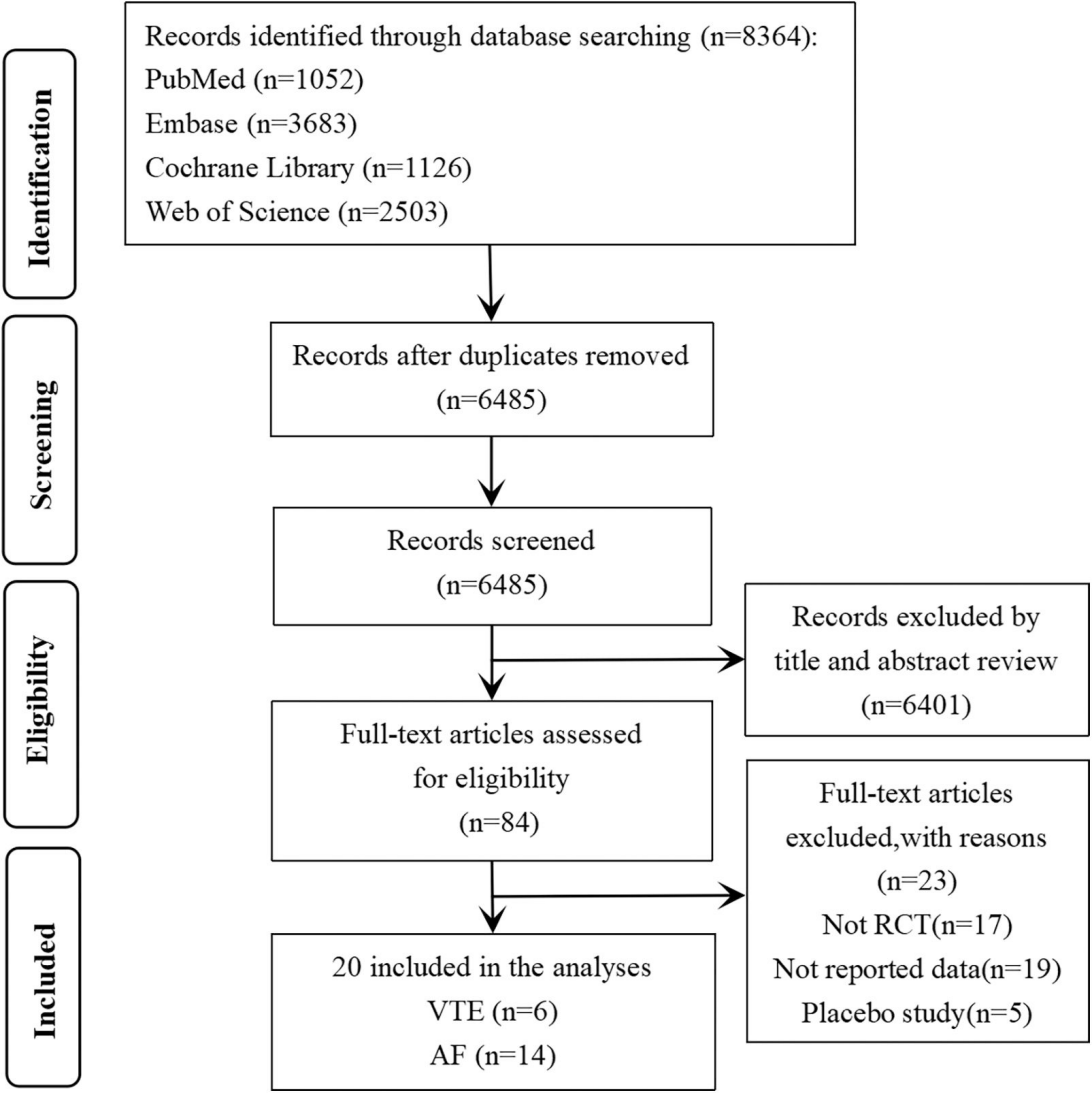
Subgroup analysis forest plot (A. Venous thromboembolism; B. Atrial fibrillation).

The flowchart of literature search is shown in Figure 1. A total of 8,364 articles were found. After literature screening, 20 RCTs that met the research requirements were finally included (9–28).

### 3.2 Baseline characteristics of included study

These 20 RCTs involved a total of 127,267 participants. 6 of them were for the treatment of VTE, and 14 were for AF. For DOACs, 4 studies included apixaban, 5 studies included dabigatran, 5 studies included edoxaban, and 6 studies included rivaroxaban. A total of 1,141 cases of ICH occurred. The baseline characteristics of the included studies are shown in Table 1.

**Table 1:** Characteristics of included studies.

	Study	Indication	DOACs	Control	Number		Age (Mean or median)		Sex (Male, %)		ICH		Follow-up
					DOACs	Control	DOACs	Control	DOACs	Control	DOACs	Control	
1	Agnelli, 2013	VTE	apixaban 10 mg twice daily for 7 days, followed by 5 mg twice daily	VKAs	2,691	2,704	57.2 (16.0)	56.7 (16.0)	58.3	59.1	3	6	6 months
2	Büller, 2012	PE	rivaroxaban 15 mg twice daily for the first 3 weeks and then 20 mg once daily	VKAs	2,412	2,405	57.9 (7.3)	57.5 (7.2)	54.1	51.7	3	13	3–12 months
3	Büller, 2013	VTE	edoxaban 60 mg once daily, or 30 mg once daily (Special populations)	VKAs	4,118	4,122	55.7 (16.3)	55.9 (16.2)	57.3	57.2	5	18	12 months
4	Schulman, 2009	VTE	dabigatran 150 mg twice daily	VKAs	1,274	1,265	55.0 (15.8)	54.4 (16.2)	58	58.9	0	3	6 months
5	Schulman, 2014	VTE	dabigatran 150 mg twice daily	VKAs	1,279	1,289	54.7 (16.2)	55.1 (16.3)	61	60.2	2	6	6 months
6	Schulman, 2013	VTE	dabigatran 150 mg twice daily	VKAs	1,430	1,426	55.4 (15.0)	53.9 (15.3)	60.9	61.1	2	4	18 months
7	Calkins, 2017	AF and ablation	dabigatran 150 mg twice daily	VKAs	317	318	59.1 (10.4)	59.3 (10.3)	72.6	77	0	2	1 week
8	Cappato, 2014	AF and cardioversion	rivaroxaban 20 mg once daily (15 mg once daily under special clinical conditions)	VKAs	988	499	64.9 (10.6)	64.7 (10.5)	72.6	73.1	2	1	30 days
9	Connolly, 2009	AF	dabigatran 110 mg or 150 mg twice daily	VKAs	6,015; 6,076	6,022	71.4 (8.6); 71.5 (8.8)	71.6 (8.6)	64.3; 63.2	63.3	27; 36	87	2.0 years
10	Giuglian, 2013	AF	edoxaban 60 or 30 mg once daily	VKAs	7,012; 7,002	7,012	72; 72	72	62.1; 61.2	62.5	61; 41	132	2.8 years
11	Granger, 2011	AF	apixaban 5 mg twice daily	VKAs	9,120	9,081	70	70	64.5	65	52	122	1.8 years
12	Hohnlose, 2019	AF and ablation	edoxaban 60 mg once daily (30 mg once daily under special clinical conditions)	VKAs	411	203	60	61	70.6	73.4	1	0	30 days
13	Hong, 2017	AF	rivaroxaban 20 mg once daily	VKAs	95	88	70.2 (10.1)	70.6 (10.9)	57.9	59.1	30	25	4 weeks
14	Hori, 2012	AF	rivaroxaban 15 mg once daily	VKAs	639	639	71	71.2	82.9	78.2	5	10	30 days
15	Kirchhof, 2018	AF and ablation	apixaban 5 mg twice daily (2.5 mg twice daily under special clinical conditions)	VKAs	318	315	64	64	69	65	0	1	3 months
16	Lopes, 2019	AF and ACS or PCI	apixaban 5 mg twice daily (2.5 mg twice daily under special clinical conditions)	VKAs	2290	2259	70.4	70.9	70.9	71.1	5	13	7 months
17	Patel, 2011	AF	rivaroxaban 20 mg once daily (15 mg once daily under special clinical conditions)	VKAs	7111	7125	73	73	60.3	60.3	55	84	707 days
18	Mieghem, 2020	AF	edoxaban 60 mg once daily	VKAs	713	713	82.1 (5.4)	82.1 (5.5)	51.3	53.6	16	21	36 months
19	Okumura, 2020	AF	edoxaban 15 mg once daily	VKAs	492	492	86.6 (4.2)	86.6 (4.2)	42.6	42.6	2	4	48 weeks
20	Connolly, 2022	AF	rivaroxaban 20 mg once daily (15 mg once daily under special clinical conditions)	VKAs	2265	2251	50.7 (14.8)	50.7 (14.8)	27.6	27.6	8	14	6 months

VTE, venous thromboembolism; DVT, deep venous thrombosis; PE, pulmonary embolism; DOACs, direct oral anticoagulants; VKA, vitamin-K antagonist; ICH, intracranial hemorrhage.

Mean (standard deviation) or median (interquartile range).

### 3.3 Risk of bias in included studies and GRADE evaluation results

**Figure 2:**
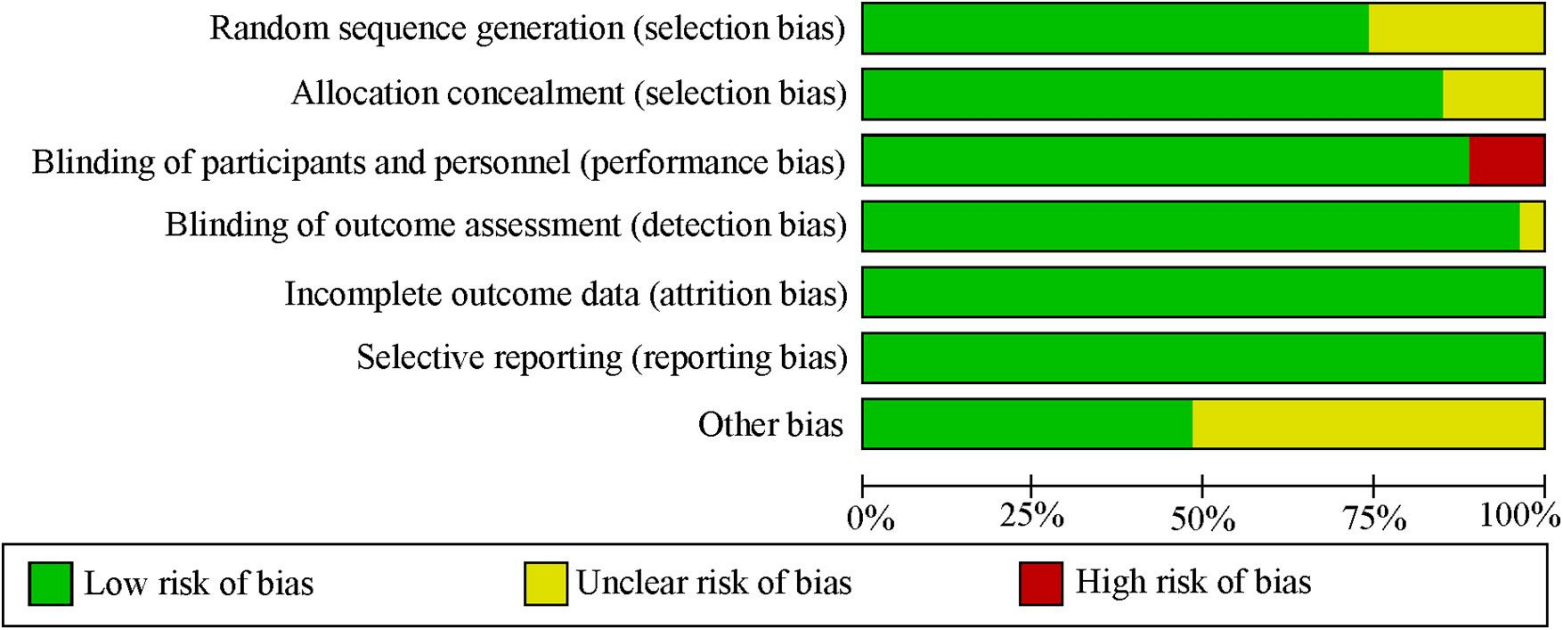
Subgroup analysis chart showing the ranking for cumulative probability **(A)** venous thromboembolism; **(B)** atrial fibrillation.

Figure 2 depicts the assessment of the risk of bias of the included studies. Overall, the risk of bias of the included RCTs was reasonable.

The GRADE evaluation results are given in Table 2. The evaluation results showed that most of the studies provided moderate evidence and the small studies provided low and high evidence, indicating that the overall level of evidence was moderate.

**Table 2:** GRADE assessment for direct and indirect comparison.

No. of studies	Certainty assessment domains						Overall certainty	Relative effect (95% CI)
	Types of Comparisons	Risk of bias	Indirect	Inconsistent	Imprecise	Publication bias		
Dabigatran vs. Apixaban								
9	Indirect effects	Serious^a^	Not serious	Not serious	Serious^b^	Undetected	㊉㊉⦻⦻ LOW	OR: 0.85; 95% CI: 0.50–1.43
Edoxaban vs. Apixaban								
9	Indirect effects	Serious^a^	Not serious	Not serious	Serious^b^	Undetected	㊉㊉⦻⦻ LOW	OR: 1.00; 95% CI: 0.62–1.63
Rivaroxaban vs. Apixaban								
10	Indirect effects	Serious^a^	Not serious	Not serious	Serious^b^	Undetected	㊉㊉⦻⦻ LOW	OR: 1.60; 95% CI: 0.96–2.68
VKAs vs. Apixaban								
4	Direct effects	Not serious	Not serious	Not serious	Not serious	Undetected	㊉㊉㊉㊉ HIGH	OR: 2.40; 95% CI: 1.62–3.56
Edoxaban vs. Dabigatran								
10	Indirect effects	Serious^a^	Not serious	Not serious	Serious^b^	Undetected	㊉㊉⦻⦻ LOW	OR: 1.18; 95% CI: 0.76–1.85
Rivaroxaban vs. Dabigatran								
11	Indirect effects	Serious^a^	Not serious	Not serious	Not serious	Undetected	㊉㊉㊉⦻ MODERATE	OR: 1.89; 95% CI: 1.18–3.04
VKAs vs. Dabigatran								
5	Direct effects	Serious^a^	Not serious	Not serious	Not serious	Undetected	㊉㊉㊉⦻ MODERATE	OR: 2.83; 95% CI: 2.00–3.99
Rivaroxaban vs. Edoxaban								
11	Indirect effects	Serious^a^	Not serious	Not serious	Not serious	Undetected	㊉㊉㊉⦻ MODERATE	OR: 1.60; 95% CI: 1.04–2.47
VKAs vs. Edoxaban								
5	Direct effects	Serious^a^	Not serious	Not serious	Not serious	Undetected	㊉㊉㊉⦻ MODERATE	OR: 2.39; 95% CI: 1.81–3.17
VKAs vs. Rivaroxaban								
6	Direct effects	Serious^a^	Not serious	Not serious	Not serious	Undetected	㊉㊉㊉⦻ MODERATE	OR: 1.50; 95% CI:1.08–2.07

GRADE Working Group grades of evidence.

High certainty: we are very confident that the true effect lies close to that of the estimate of the effect.

Moderate certainty: we are moderately confident in the effect estimate: the true effect is likely to be close to the estimate of the effect, but there is a possibility that it is substantially different.

Low certainty: our confidence in the effect estimate is limited: the true effect may be substantially different from the estimate of the effect.

Very low certainty: we have very little confidence in the effect estimate: the true effect is likely to be substantially different from the estimate of effect.

Explanations.

a Risk of bias: Dominated by evidence at high or moderate risk of bias.

b Imprecision: Confidence intervals include values favouring either treatment.

### 3.4 Network evidence plot and inconsistency analysis

**Figure 3:**
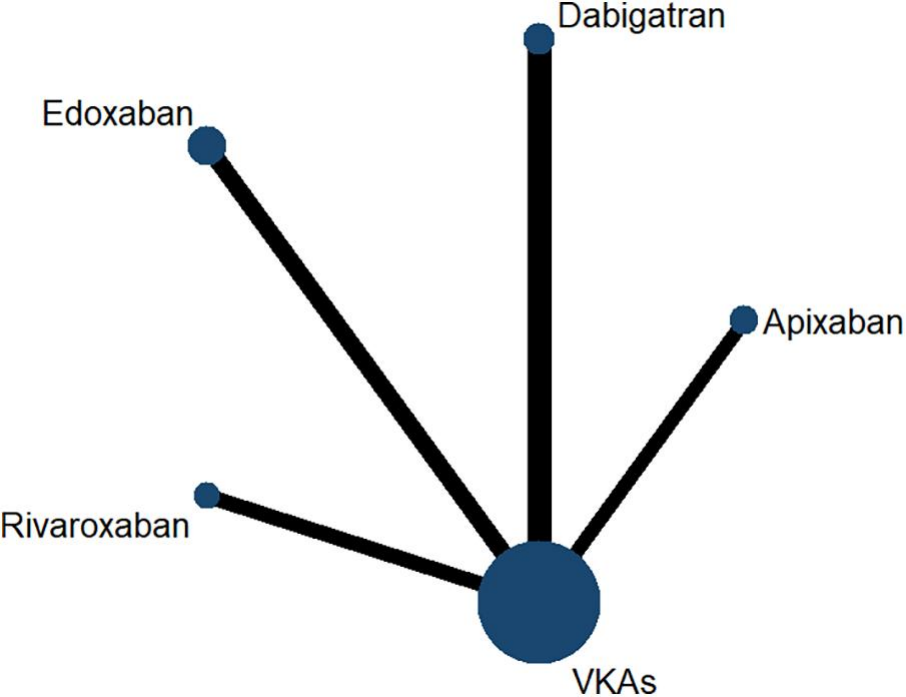
Network evidence map of all included studies.

The network included five different interventions, namely apixaban, dabigatran, edoxaban, rivaroxaban, and VKAs. Figure 3 shows the network evidence plot among the interventions. A straight line connection between the points indicates that there was a direct comparative study between different interventions. The size of each node represents the sample size of the intervention, and the thickness of each line segment represents the number of studies between interventions. Our current evidence network does not form a closed loop. This is because there are currently no randomized controlled trials comparing different DOACs directly, which is an objective reflection of the current state of evidence in this field. Due to the lack of direct comparisons, the results are based only on indirect evidence, and the uncertainty is high. Therefore, we will support the rationality of the indirect comparisons through the transitivity assumption.

### 3.5 Assessment of transitivity assumption

Although we demonstrated through a detailed feature comparison table that all the trials had overall similarities in key demographic, clinical characteristics, and intervention protocols, some differences still existed. For instance, the average follow-up time varied significantly among the trials, which might affect the cumulative rate of ICH events. Additionally, although we combined patients with AF and VTE, these two groups had inherent differences in age, comorbidities, and anticoagulation intensity. Although we used a random effects model to combine the data and conducted subgroup analyses when possible, these differences in population and design remain potential sources of bias, which may affect the accuracy of indirect comparisons. Therefore, when interpreting comparisons between different DOACs, especially when the effect estimates are similar, caution should be exercised.

### 3.6 Network meta-analysis outcome

**Figure 4:**
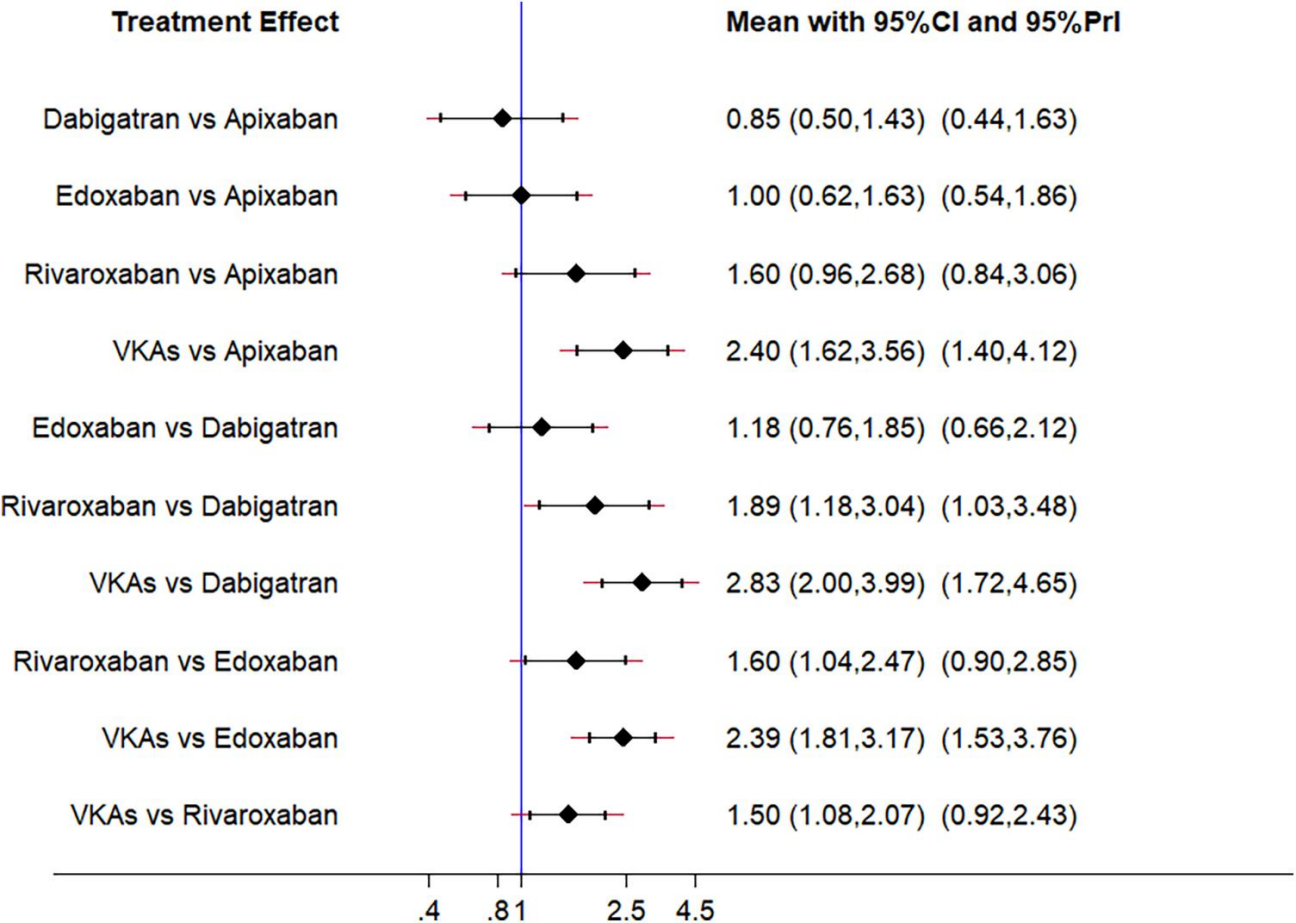
Forest plots of intracranial hemorrhage.

NMA results for ICH are shown in Figure 4. Compared with apixaban, VKAs (OR: 2.40, 95% CI: 1.62–3.56) had a higher risk of bleeding, and the difference was significant. Compared with dabigatran, rivaroxaban (OR: 1.89, 95% CI: 1.18–3.04) and VKAs (OR: 2.83, 95% CI: 2.00–3.99) had a higher risk of bleeding, and the difference was significant. Compared with edoxaban, rivaroxaban (OR: 1.60, 95% CI: 1.04–2.47) and VKAs (OR: 2.39; 95% CI: 1.81–3.17) had a higher risk of bleeding, and the difference was significant. Compared with rivaroxaban, VKAs (OR: 1.50; 95% CI: 1.08–2.07) had a higher risk of bleeding, and the difference was significant.

**Figure 5:**
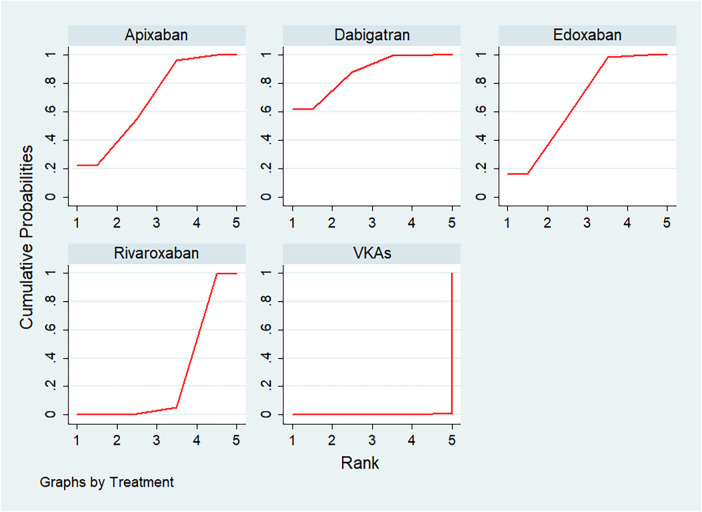
Cumulative probability ranking chart of intracranial hemorrhage.

In the ranking of the cumulative probability of ICH (Figure 5), dabigatran (SUCRA 87.6) had the highest safety, followed by apixaban (SUCRA 68.6), edoxaban (SUCRA 67.5), rivaroxaban (SUCRA 26.1), and VKAs (SUCRA 0.2).

### 3.7 Publication bias

**Figure 6:**
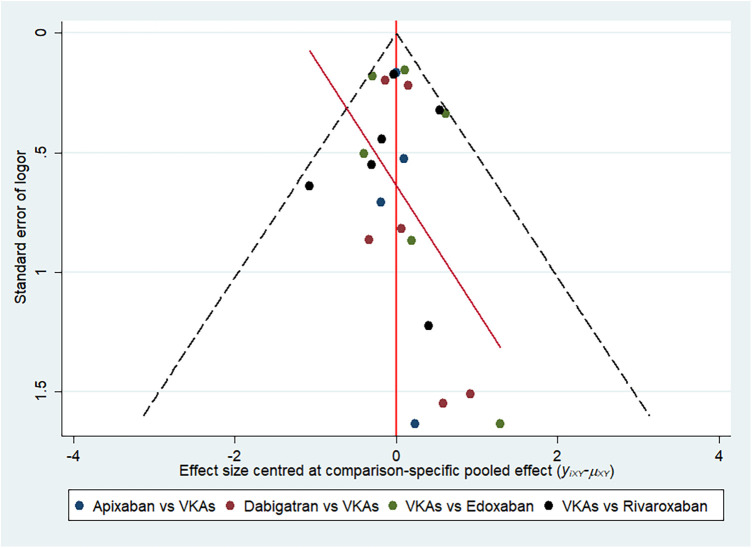
Funnel plots of all included studies.

Funnel charts and Egger test *p* values were generated to evaluate the publication bias of included studies (Figure 6). The scattered points were basically distributed in a funnel symmetrical manner and the *p* value was >.05, which meant there may be no significant publication bias. However, this result should be interpreted with caution, and it cannot completely rule out the possibility of the existence of small sample study effects.

### 3.8 Subgroup meta-analysis outcome

To explore the impact of different indications on the bleeding risk of anticoagulants, we conducted subgroup analyses based on the indications (in Supplementary Figures 1, 2). In the VTE group, the SUCRA values were ranked as follows: rivaroxaban (SUCRA 76.7), edoxaban (SUCRA 69.5), dabigatran (SUCRA 57.8), apixaban (SUCRA 40.8) and VKAs (SUCRA 5.3). Compared with edoxaban, VKAs (OR: 3.61, 95% CI: 1.34–9.73) had a higher risk of bleeding, and the difference was significant. Compared with rivaroxaban, VKAs (OR: 4.36, 95% CI: 1.24–15.33) had a higher risk of bleeding, and the difference was significant. In the AF group, the SUCRA values were ranked as follows: dabigatran (SUCRA 86.8), apixaban (SUCRA 71.8), edoxaban (SUCRA 65.2), rivaroxaban (SUCRA 25.4), and VKAs (SUCRA 0.9). Compared with apixaban, VKAs (OR: 2.44, 95% CI: 1.57–3.79) had a higher risk of bleeding, and the difference was significant. Compared with dabigatran, rivaroxaban (OR: 2.04, 95% CI: 1.21–3.44) and VKAs (OR: 2.83, 95% CI: 1.93–4.15) had a higher risk of bleeding, and the difference was significant. Compared with edoxaban, rivaroxaban (OR: 1.66, 95% CI: 1.03–2.66) and VKAs (OR: 2.30, 95% CI: 1.67–3.17) had a higher risk of bleeding, and the difference was significant.

## 4 Discussion

This study included 20 RCTs, and an NMA was used to independently compare the risks of ICH caused by DOACs used for treating VTE and AF. In the ranking of the cumulative probability of ICH, dabigatran had the highest safety, followed by apixaban, edoxaban, rivaroxaban, and VKAs. Compared with VKAs, apixaban, dabigatran, edoxaban, and rivaroxaban all had a lower risk of bleeding, and the difference was significant. Compared with rivaroxaban, dabigatran and edoxaban had a lower risk of bleeding, and the difference was significant.

ICH is the most serious complication of anticoagulant therapy in patients. It can lead to a decline in patient health and can increase patient mortality and disability (29–31). This study confirmed that compared with VKAs, all four DOACs can significantly reduce the risk of ICH. This conclusion is consistent with previous studies. In the ranking of the ICH risk of DOACs, rivaroxaban has the highest risk, while dabigatran has the lowest. This result is in line with the report of Wolfe et al. (32). The underlying mechanism may be related to the characteristics of drug interactions: dabigatran, as a direct thrombin inhibitor, is only a substrate of P-glycoprotein (P-gp); while direct Xa factor inhibitors such as rivaroxaban, apixaban, and edoxaban are also substrates of cytochrome P450 enzymes and P-gp. Moreover, rivaroxaban is also a substrate of the breast cancer resistance protein (33). Its more complex metabolic pathway may enhance drug interactions, thereby affecting its pharmacokinetic and pharmacodynamic characteristics, which may be one of the reasons for its relatively higher risk of ICH.

Wolfe et al. (32) found that dabigatran reduced the risk of ICH by 70%, edoxaban by 63%, apixaban by 54%, and rivaroxaban by 31% compared with VKAs through the NMA (17 RCTs, including patients with AF, VTE and VTE prophylaxis), this is similar to our result. This meta-analysis was published in 2018. It has been eight years since then. During this period, many new RCTs have been published, and this study did not conduct subgroup analysis on indication. Therefore, it is necessary to incorporate the new RCTs and conduct subgroup analyses based on different indications. This will update the meta-analysis. This will update the meta-analysis. As new studies continue to be published, timely updates can incorporate the latest data, making the results more accurate and verifying the stability of previous conclusions.

However, in another NMA (34), the risk of ICH for rivaroxaban was the lowest among the DOACs, which clearly contradicts our research results. This study only included VTE patients and did not include AF patients, which might be the main reason for the different results. This suggests that different disease states may have different bleeding risks. Therefore, independent analysis of VTE and AF is crucial.

This NMA provides the latest evidence at the population level for the advantages of DOACs over VKAs in reducing the risk of ICH. However, translating this population-level evidence into individual patient clinical decisions requires consideration of the complexity of bleeding risk. The risk of ICH is not solely determined by the type of anticoagulant drug; it may also be the result of the interaction between the drug characteristics and specific patient factors (such as age, liver function, concomitant medications, and genetic background). It is worth noting that emerging evidence indicates that even modifiable biomarkers, such as low-density lipoprotein cholesterol levels, are independently associated with the bleeding risk of anticoagulant-treated patients (35). This suggests that future anticoagulation management should follow a stratification strategy: first, based on this study and similar evidence, select the anticoagulant drug category (DOACs) and specific brand that offers a better risk profile for ICH for the patient; then, the drug selection must be incorporated into a broader individualized risk assessment framework, considering all relevant risk factors for the patient.

This study observed that there are differences in the risk of ICH among different DOACs, and the underlying mechanism is worthy of exploration. Besides the differential impact on hemostatic potential caused by the different targets of dabigatran (a direct thrombin inhibitor) and other DOACs (Xa factor inhibitors), a more systematic biological perspective may provide supplementary explanations. Thrombosis and hemorrhage involve a dynamic balance of endothelial integrity, platelet function, inflammation, and fibrinolytic systems (36). Therefore, we hypothesize that the differences in bleeding risk among different DOACs may be partially due to their differential “multifaceted” regulatory effects on these extensive vascular biological pathways, such as different impacts on endothelial cell function or local inflammatory responses. This hypothesis awaits direct verification through future basic research and may provide new ideas for optimizing the safety of anticoagulant therapy.

In this study, patients with AF and VTE were combined to analyze the risk of ICH associated with DOACs. The main considerations were as follows: Firstly, when clinicians encounter a newly diagnosed patient requiring anticoagulation, regardless of whether the indication is AF or VTE, they need to make a choice among similar drugs (such as different DOACs). A comprehensive, cross-indication safety comparison map can provide more efficient and macroscopic references. Secondly, the accuracy of the results of the NMA is highly related to the number of RCTs. Combining two large patient populations increases the accuracy of the comparison, especially in cases of rare but severe events such as ICH. Thirdly, regardless of the indication, the mechanism and pharmacological targets of DOACs are the same. Of course, the combined analysis of indications does introduce some heterogeneity. We did not ignore this point but actively conducted subgroup analyses by indication in the analysis. The relative ranking of the drugs in terms of bleeding risk for different indications is one of the core exploration issues of this study.

To clarify the impact of different clinical indications on the risk of bleeding, this study conducted subgroup analyses, conducting statistical evaluations separately for the AF and VTE patient groups, and obtained the specific bleeding risk ranking results based on the indications. In the AF group, the ranking of the SUCRA values remained unchanged, which was consistent with the previous main results. In the VTE group, the ranking of SUCRA values changed. Rivaroxaban had the lowest bleeding risk, while apixaban had the highest. The differences in subgroup results are likely due to the systematic differences in the pathophysiological background, treatment intensity, comorbidities, and concomitant medications between the two types of diseases. The differences in subgroup results suggest that the ranking of bleeding risks for DOACs should not be simply extrapolated across different indications. The drug with the lowest risk of ICH in AF may not be the best overall in terms of bleeding risk in VTE. This difference highlights the need for clinical decisions to be based on high-level evidence specific to the indications. Further exploration of the mechanisms underlying this difference is required to optimize individualized anticoagulation strategies in the future.

The results of this study are derived from strictly designed randomized controlled trials, which usually have clear inclusion and exclusion criteria. This may limit their representativeness for a broader and more heterogeneous clinical population. In real-world clinical practice, the risk of bleeding and thrombosis in anticoagulant patients is strongly influenced by age, frailty, burden of comorbidities (such as liver and kidney dysfunction), and polypharmacy (37). For instance, advanced age and frailty are not only independent risk factors for bleeding but also may alter the pharmacokinetics of drugs, thereby affecting the relative safety of different DOACs. Although we conducted subgroup analyses of AF and VTE, data for the specific high-risk subgroups (such as extremely frail elderly individuals and those with severe renal dysfunction) are still insufficient. Therefore, when directly extrapolating the population-level conclusions of this study to all patients, caution is necessary. For elderly or complex patients with multiple comorbidities, clinical decisions should be more individualized. While referring to the relative risk differences provided by this study, comprehensive assessment of the patient's overall condition, life expectancy, risk of falls, and specific concomitant medications must also be conducted. Future research should aim to verify the efficacy and safety of different DOACs in these key subgroups in more real-world observational data or individual-level meta-analyses of patients.

This study has the following advantages: Firstly, to our knowledge, this NMA is currently the most comprehensive study in this field, as we have carefully compared the risk of ICH of DOACs and VKAs in patients with AF and VTE, and sorted them based on safety. Secondly, this study included AF patients with different clinical conditions and conducted subgroup analyses for these different clinical conditions, which may have certain reference value for patients with different clinical situations. Thirdly, since the use of unconventional doses may have limited clinical guiding significance and may lead to result bias, our study only included studies using conventional doses, and these studies may be more valuable for clinical practice.

However, there are certain limitations inherent to any meta analysis. Firstly, combining patients from different anticoagulation indication groups may introduce clinical heterogeneity. Although subgroup analyses have been conducted, the differences in patient baseline characteristics and treatment goals still need to be taken into account as background factors when interpreting the results. Secondly, the commonly used doses of DOACs have not been subdivided, and there are differences in bleeding risks among different doses of the same drug. It will be necessary in the future to subdivide the doses in order to assess their safety and provide more accurate evidence. Thirdly, the network structure does not contain closed loops, meaning that all DOACs are only directly compared with VKAs, while there is a lack of head-to-head direct evidence among DOACs. Although this structure is common in such analyses, it limits our ability to conduct formal statistical inconsistency tests. Despite our support for the rationality of these comparisons through strict transitivity assessment, the absence of direct evidence means that the uncertainty of these indirect comparison results is relatively high, and particular caution should be exercised when interpreting the subtle differences among DOACs.

## 5 Conclusions

All DOACs had a lower risk of ICH than VKAs. Dabigatran may be the safest choice among any anticoagulant regarding risk of ICH.

## Data Availability

The raw data supporting the conclusions of this article will be made available by the authors, without undue reservation.
