# Supplementary material for: Risk of intracranial hemorrhage with direct oral anticoagulants: an updated network meta-analysis of randomized controlled trials
Source: Front Cardiovasc Med. 2026 May 19;13:1835091. doi: 10.3389/fcvm.2026.1835091 (PMC13226517; doi:10.3389/fcvm.2026.1835091)
Supplement: Supplementary file 1 [file Supplementaryfile1.docx]

**Supplementary Materials**

**Figure 1. Subgroup analysis forest plot (A. Venous thromboembolism; B. Atrial fibrillation)**

**B**

**A**


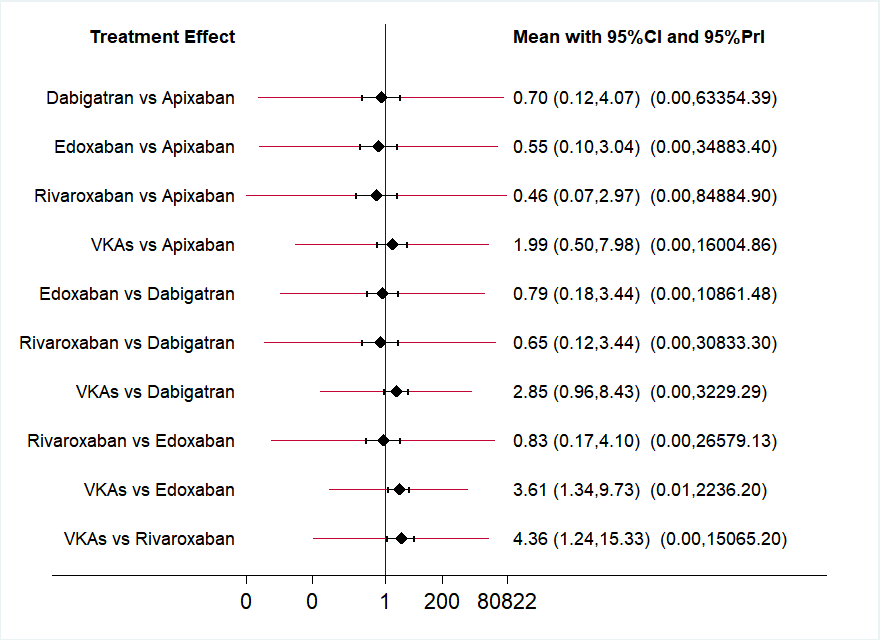

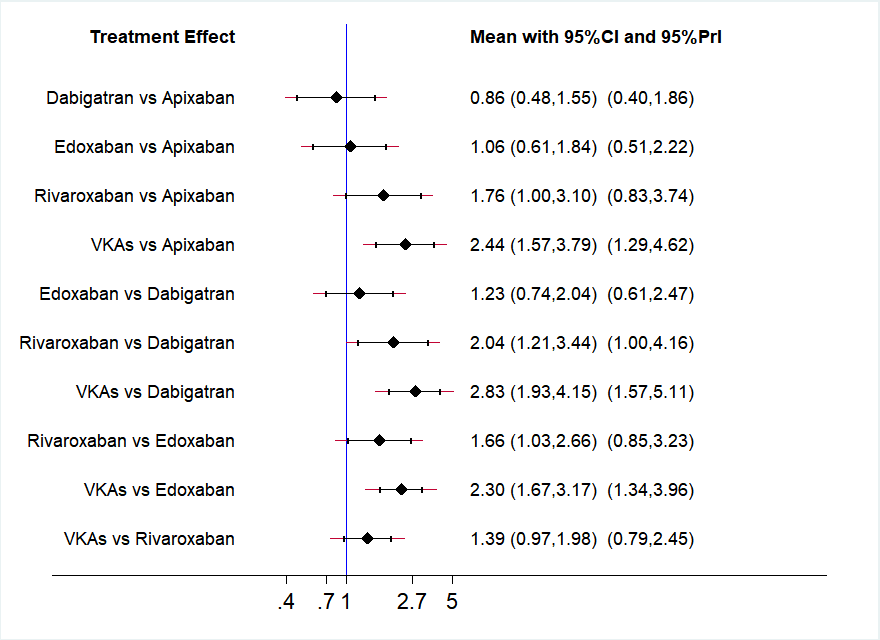


**Figure 2. Subgroup analysis Chart showing the ranking for cumulative probability (A. Venous thromboembolism; B. Atrial fibrillation)**

**B**


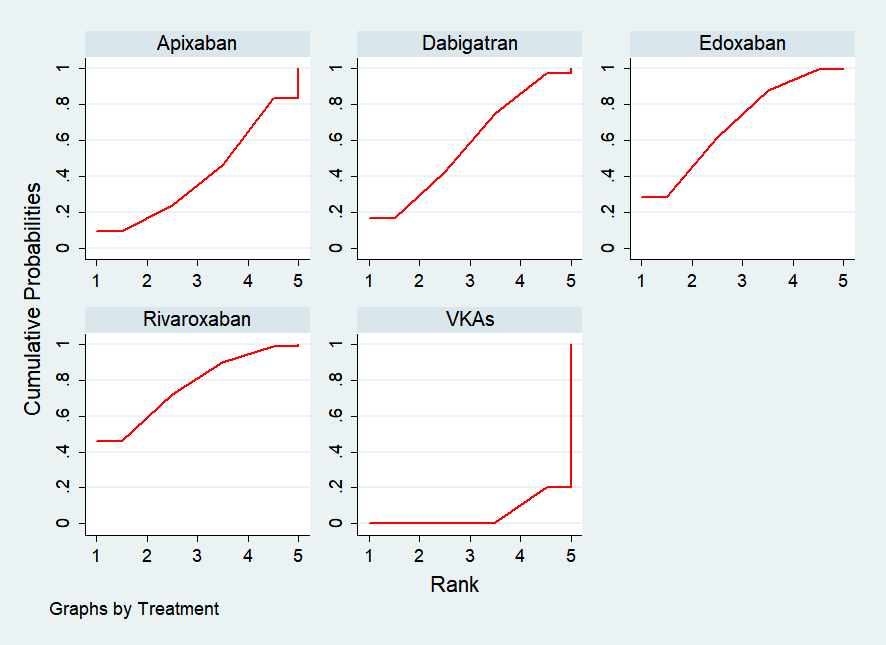

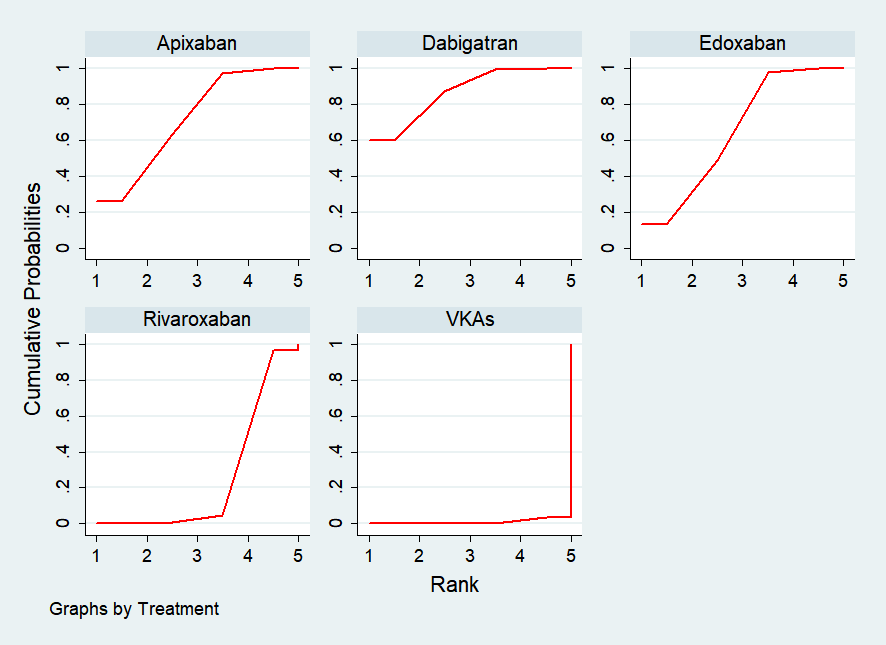


**A**
